# ‘Getting back to normal’: using patients’ lived experience to inform a core outcome set for cardiac arrest clinical trials

**DOI:** 10.1186/1745-6215-16-S3-P3

**Published:** 2015-11-24

**Authors:** Laura Whitehead, Gavin Perkins, Kirstie Haywood

**Affiliations:** 1 Division of Health Sciences, Warwick Medical School, University of Warwick, Coventry, UK; 2 Warwick Clinical Trials Unit, Warwick Medical School, University of Warwick, Coventry, UK; 3 Royal College of Nursing Research Institute, Warwick Medical School, University of Warwick, Coventry, UK; 4 Heart of England NHS Foundation Trust, Birmingham, UK

## Background

A recent review of outcome reporting in out of hospital cardiac arrest (OHCA) clinical trials detailed a lack of transparency and heterogeneity, highlighting the need to establish a core outcome set (COS)(Whitehead et al., 2015). Moreover, the review highlighted the dominance of outcomes which focused on the pathophysiological manifestations of the event and clinicians’ perspectives, with an absence of outcomes which sought to explore the patients’ perspective. To ensure the development of a COS with relevance and meaning to all stakeholders - patients, clinicians and researchers, we sought to explore the views of patients and their partners to improve our understanding of the outcomes that really matter.

## Methods

Semi-structured interviews were conducted with OHCA patients and, where possible their partners separately to gain a better understanding of the patients’ experience. Participants were recruited from a large NHS trust in the West Midlands, UK. Inclusion criteria included aged >18 years, cognitively able, and not critically ill. An interpretative phenomenological analysis was adopted.

## Results

A convenience sample of eight patients (62.8 years (SD 13.6); range 41-79; n=5 male (62.5%) and three of their partners were interviewed, between 3 and 12 months post-arrest. At the time of interviews 2 had returned to work, 1 was planning a phased return to work, 4 were previously retired and 1 was previously unemployed. Analysis highlighted an overarching theme of “disruption to normality” with patients’ expressing a strong desire to get back to normal. The superordinate themes which contribute to this disruption include: survival, physical function, emotional well-being, social well-being and participation and the impact on others.

## Conclusions

This study details an exploration of the lived experience of the survivors of cardiac arrest and their partners. It provides clear, patient-derived guidance for the health outcomes that matter to patients and which should be considered for COS inclusion.

